# Faecal occult blood screening and reduction of colorectal cancer mortality: a case-control study

**DOI:** 10.1038/sj.bjc.6690107

**Published:** 1999-02

**Authors:** J Faivre, M A Tazi, T ElMrini, C Lejeune, A M Benhamiche, F Dassonville

**Affiliations:** Registre Bourguignon des Cancers Digestifs (Equipe associée INSERM-DGS and INSERM CRI 95 05), Faculté de Médecine, Dijon Cedex, 21033, France

**Keywords:** colorectal cancer, case–control study, screening, faecal occult blood test

## Abstract

To estimate the efficacy of screening on colorectal cancer mortality, a population-based case–control study was conducted in well-defined areas of Burgundy (France). Screening by faecal occult blood test prior to diagnosis in cases born between 1914 and 1943 and who died of colorectal cancer diagnosed in 1988–94 was compared with screening in controls matched with the case for age, sex and place of residence. Cases were less likely to have been screened than controls, with an odds ratio (OR) of 0.67 [95% confidence interval (CI) 0.48–0.94]. The negative overall association did not differ by gender or by anatomical location. The odds ratio of death from colorectal cancer was 0.64 (95% CI 0.46–0.91) for those screened within 3 years of case diagnosis compared with those not screened. It was 1.14 (95% CI 0.50–2.63) for those screened more than 3 years before case diagnosis. There was a negative association between the risk of death from colorectal cancer and the number of participations in the screening campaigns. The inverse association between screening for faecal occult blood and fatal colorectal cancer suggests that screening can reduce colorectal cancer mortality. This report further supports recommendations for population-based mass screening with faecal occult blood test. © 1999 Cancer Research Campaign


*British Journal of Cancer* (1999) **79**(3/4), 680–683
© 1999 Cancer Research Campaign
Article no. bjoc.1998.0107

### Faecal occult blood screening and reduction of colorectal cancer mortality: a case-control study

**J Faivre, MA Tazi, T El Mrini, C Lejeune, AM Benhamiche and F Dassonville**

Registre Bourguignon des Cancers Digestifs (Equipe associée INSERM-DGS and INSERM CRI 95 05), Faculté de Médecine, 7 Boulevard Jeanne D'Arc, 21033, Dijon Cedex, France

**Summary** To estimate the efficacy of screening on colorectal cancer mortality, a population-based case–control study was conducted in well-defined areas of Burgundy (France). Screening by faecal occult blood test prior to diagnosis in cases born between 1914 and 1943 and who died of colorectal cancer diagnosed in 1988–94 was compared with screening in controls matched with the case for age, sex and place of residence. Cases were less likely to have been screened than controls, with an odds ratio (OR) of 0.67 [95% confidence interval (CI) 0.48–0.94]. The negative overall association did not differ by gender or by anatomical location. The odds ratio of death from colorectal cancer was 0.64 (95% CI 0.46–0.91) for those screened within 3 years of case diagnosis compared with those not screened. It was 1.14 (95% CI 0.50–2.63) for those screened more than 3 years before case diagnosis. There was a negative association between the risk of death from colorectal cancer and the number of participations in the screening campaigns. The inverse association between screening for faecal occult blood and fatal colorectal cancer suggests that screening can reduce colorectal cancer mortality. This report further supports recommendations for population-based mass screening with faecal occult blood test.

**Keywords:** colorectal cancer; case–control study; screening; faecal occult blood test

### INTRODUCTION

The attempted reduction of colorectal cancer (CRC) mortality is an issue of great importance. It is the second most frequent cancer in men and women in Europe and North America (Parkin et al, 1997). Despite advances in diagnostic techniques and treatment, the 5-year survival rate remains poor (Berrino et al, 1995). Three randomized studies have indicated that faecal occult blood testing (FOBT) followed, in the event of a positive test, by a colonoscopy to detect the source of bleeding can reduce CRC mortality (Kronborg et al, 1996; Hardcastle et al, 1996; Mandel et al, 1993). The results of one of these studies, conducted in volunteers, with a high positivity rate of rehydrated FOBT, are not applicable to a general population (Mandel et al, 1993). In particular, it is not possible to perform a colonoscopy on as much as nearly 10% of the population at each screening round. The two population-based studies from Denmark and England indicate that biennial screening with FOBT can reduce CRC mortality by 15–18%. It is not known whether the results of these studies can be extrapolated to other countries (such as France) where the prognosis of CRC in the general population is better than in Denmark or England (Berrino et al, 1995). In this situation, the reduction in CRC mortality due to FOBT might be lower and a population-based controlled study of the question is under way in Burgundy (Tazi et al, 1997). The final results will be available within 2 years. In order to provide an interim evaluation of efficacy of the programme, we conducted a case–control study within the enrolled arm of the population study, comparing participants with non-participants to evaluate the effect of screening on mortality from colorectal cancer, taking into consideration the relationship with the interval since the most recent screening test and the number of screenings.

*Received 13 May 1998*
*Revised 29 June 1998*
*Accepted 8 July 1998*

*Correspondence to:* J Faivre

### MATERIALS AND METHODS

### Study setting

The design of the study has been reported previously (Tazi et al, 1997). Briefly, faecal occult blood screening using the Hemoccult II test was offered to all the residents of 12 well-defined administrative areas of Burgundy (France) who were born between 1914 and 1943. The enrolled population was 45 642. The first screening campaign began in 1988 and, for part of the study population, in 1989. It was repeated in 1990, 1992 and 1994. No other method of screening for CRC was offered to this population. The screening test was provided free of charge by primary care physicians over a 4-month period, then posted to persons who had not consulted a general practitioner during this period, with a reminder letter after a month to non-responders. A colonoscopy was offered if the test was positive. The entire enrolled population was invited to participate in each screening campaign.

### Identification of cases

Eligible cases were those who were residents of areas covered by the screening programme, with a colorectal adenocarcinoma diagnosed between 1988 or 1989 (according to the year of inclusion into the programme) and 1995, who subsequently died before December 1996. Cases were identified either from files of the population-based cancer registry covering the study population, or through the data collection system of the screening programme. Information on method of diagnosis, subsite, histological type, treatment and stage at diagnosis was verified by reviewing all available medical records. Details of death were ascertained from death certificates, cancer registry information, local registers of births, marriages and deaths and from general practitioners. There was a total of 214 potentially eligible cases. Death was attributed to colorectal cancer if advanced or metastatic colorectal cancer was present at the time of death or if the case died in the post-operative phase. In the few instances in which the cause of death could not be easily determined, it was decided by an expert physician and a pathologist external to the study. Classification of the cause of death was carried out blind to screening history. The 36 ineligible subjects included nine whose death was due to another cancer (4.2%), four who had a second cancer and in whom the cause of death could not be determined (1.9%), two who had a non-adenocarcinoma colorectal cancer (0.9%) and 21 who died from other causes (9.8%). A total of 178 cases remained available for analysis (84% of potentially eligible cases). None of these cases was known to have a previous history of colorectal cancer, familial polyposis coli, ulcerative colitis or Crohn's disease before colorectal cancer was diagnosed.

### Identification of controls

For each case, four controls were randomly selected from the enrolled population, and matched for sex, year of birth and place of residence. Controls had to be alive when their matched case died. In the event of death, the control was replaced. Each control in the matched set thus had the same opportunity as the corresponding case of undergoing a screening test. A previous history of adenoma or non-fatal colorectal cancer first occurring after the date of diagnosis of the corresponding case was not grounds for exclusion.

### Statistical analysis

The date of diagnosis of the case was applied to the case–control matched set as the reference date, so as to ensure comparable intervals for the screening history of cases and controls. The screening history was taken from the time screening started to (and including) the date of the case diagnosis, which was determined from the screening campaign's data files. Eight or nine years of data were available for all the case–control sets according to the year of inclusion (1988 or 1989). In nine cases, it was specified that the FOBT was used as a diagnostic test and not as a screening test. These tests were not included in the analysis.

A conditional logistic regression for matched sets was used to estimate the efficacy of FOBT screening in reducing mortality from colorectal cancer. The 'no screening' reference category comprised subjects who had never participated at all. Efficacy is suggested if a screening history is more common among controls than cases. Separate ORs were also calculated by gender and subsite (right colon, left colon and rectum). Interaction terms for gender and subsite were tested for statistical significance. To address the question of an optimal screening interval, ORs for death from colorectal cancer were calculated for a history of faecal occult blood screening at different time intervals before the reference date compared with 'those never screened'. Odds ratios for death from colorectal cancer were also calculated by the number of participations in the screening campaigns, 'those never screened' being the reference class. The 95% CIs are presented for the estimated ORs. Analyses were carried out using EGRET (1993, Statistics and Epidemiology Research Corporation, Seattle, WA, USA).

### RESULTS

In the four successive rounds of screening, compliance was 52.8%, 54.0%, 57.3% and 58.3%. Overall, 68.7% of the initially invited population was given at least one screening test. Among the persons enrolled in the fourth screening campaign, 50.8% participated in all four rounds.

A significantly smaller proportion of cases than of controls underwent screening by FOBT during the study period. The OR of subjects having been exposed to one or more FOBT during the whole study period was 0.67 (95% CI 0.48–0.94) compared with persons never screened. The negative overall association did not differ significantly by gender (OR = 0.63%, 95% CI, 0.41–0.96, for men; OR = 0.74, 95% CI 0.42–1.30, for women; $P = 0.59$) or by cancer localization ($P = 0.92$). The magnitude of the mortality reduction was slightly greater for left colon cancer (OR = 0.59, 95% CI 0.39–1.06) than for right colon cancer (OR = 0.76, 95% CI, 0.39–1.51) or rectal cancer (OR = 0.71, 95% CI 0.41–1.21).

Table 1 shows the screening frequencies by the interval since the most recent FOBT for cases and controls. A history of

**Table 1** Odds ratio of death from colorectal cancer according to number of months since the most recent screening FOBT[a] (conditional logistic regression model)

| Time since last screening FOBT (years) | No. of subjects screened (%)[b] | | | |
|---|---|---|---|---|
| | Cases | Controls | Matched OR[c] (95% CI) | *P* |
| Never screened | 86 (48.3) | 277 (38.9) | 1 | < 0.001 |
| 1–3 months | 26 (14.6) | 53 (7.4) | 2.02 (1.12–3.65) | |
| 4–12 months | 29 (16.3) | 154 (21.6) | 0.58 (0.34–0.99) | |
| 13–24 months | 25 (14.0) | 173 (24.3) | 0.40 (0.23–0.68) | |
| 25–36 months | 3 (1.7) | 30 (4.2) | 0.32 (0.10–1.10) | |
| 37–48 months | 3 (1.7) | 11 (1.5) | 0.84 (0.22–3.22) | |
| 49–60 months | 1 (0.6) | 7 (1.0) | 0.41 (0.05–3.72) | |
| > 60 months | 5 (2.8) | 7 (1.0) | 2.09 (0.61–7.14) | |

[a]FOBT, faecal occult blood test. [b]Number (and percentage) of subjects who had the most recent screening test during each period of time. [c]For each line we calculate the odds ratio of being exposed to the most recent screening test during the specified period of time (compared with no FOBT).

*© Cancer Research Campaign 1999* 

screening with FOBT within 3 months of the reference date was more common among cases than among controls. This finding was reversed in the 4–12 to 25–36 month intervals, with controls more likely to have been screened than cases. The OR increased towards 1 as the interval between the test and the diagnosis lengthened. Consideration of screening intervals of 0–3 years and > 3 years gave an OR for a screening FOBT of, respectively, 0.64 (95% CI 0.46–0.91) and 1.14 (95% CI 0.50–2.63).

We observed a negative association between the risk of death from colorectal cancer and the number of screenings ($P = 0.003$, Table 2). The risk decreased significantly for those who participated in two or more screening campaigns, compared with non-participants (OR = 0.46, 95% CI 0.31–0.75). The mortality reduction is estimated to be 70% among those who participated in all four screening campaigns (OR = 0.30, 95% CI 0.12–0.76).

### DISCUSSION

This case–control study suggests a 33% reduction in colorectal cancer mortality for individuals screened by FOBT biennially. The main advantages of this study are the opportunity of including all deaths from colorectal cancer in a defined population and the holding of information of similar quality on the history of participation in screening campaigns, for both cases and controls. Matching of cases and controls by sex and birth year as well as place of residence will have minimized the effect of sociodemographic and lifestyle risk factors that could have spuriously exaggerated the estimates of efficacy. Prior screening can be a confounding factor in such studies but, before 1988, no cancer screening programme existed in the study area and FOBT was not available for individuals. Cases and controls were unlikely, therefore, to have been screened previously. Biases towards an apparent benefit of screening might operate when individuals with a lower risk of developing colorectal cancer are more likely to attend screening. Through the population-based cancer registry, data are available on the occurrence of colorectal cancer in non-participants of screening. Preliminary data indicate little difference in the incidence of colorectal cancer between non-participants and the control arm of the population studies. It is thus unlikely that selection bias of this type accounts for a substantial part of the reported effect of screening. However, the difficulty of controlling for all relevant confounding factors could still limit the accuracy of the study.

Our findings are consistent with previous case–control studies using the same FOBT, the Hemoccult test. Two case–control studies conducted among members of health insurance plans on the west coast of the United States and a population-based study in the district of Florence, Italy, suggest a reduction in colorectal cancer mortality of 28% (Lazovich et al, 1995), 25% (Selby et al, 1993) and 40% (Zappa et al, 1997) for people who performed a Hemoccult test. In a German study, in which results were reported by sex, mortality reduction was 57% for females and 8% for males for those screened 6–36 months before the reference date (Warhendorf et al, 1993). In one case–control study, no evidence of efficacy was found (Newcomb et al, 1992). However, this study was very small, with only 66 cases, and the analysis was conducted without regard to the period of screening. Compared with the other studies, mortality reduction was higher in a Japanese case–control study using an immunochemical haemagglutination test annually (Saito et al, 1995). This result is easily explained by the higher sensitivity of this test. However, its specificity is not well established at a population level (probably lower than the specificity of the Hemoccult test) and the test is more expensive and more difficult to analyse and thus is not as suitable for a mass screening programme.

The results of this case–control study also appear to be consistent with true effectiveness, as reported by population-based randomized trials (Hardcastle et al, 1996; Kronborg et al, 1996), which found a colorectal cancer mortality reduction ranging between 18% and 15%. As case–control studies compare participants with non-participants, they provide an indication of reduction in risk, that is independent of compliance rates. In randomized studies, as in the general population, compliance rates affect the final result. With a 50% compliance rate, true effectiveness in the present study would be half of the mortality reduction reported.

None of the studies, except one, report a difference between males and females for a protective effect of FOBT. The reason for the discrepancy seen in Saarland has not been explained (Warhendorf et al, 1993). As found in the present study, it has been previously reported that the protective effect of screening is of the same order of magnitude whatever the anatomical location (Selby et al, 1993; Saito et al, 1995). The slightly lower OR for sigmoid and descending colon can be attributed to a higher sensitivity of FOBT for these cancers (Thomas et al, 1992). It is much lower for rectal cancers. Surprisingly, the Italian case–control study suggests a higher reduction in risk for rectal cancer than for colon cancer (Zappa et al, 1997). As expected we found that cases (persons with fatal colorectal cancer) were more likely than controls to have been screened in the 3 months before the reference date. It is easy to understand that more cases than controls have undergone a

**Table 2** Odds ratio of death from colorectal cancer according to number of participations before the reference date (conditional logistic regression model)

| No. of participations in the screening campaigns | No. of subjects (%)[a] Cases (*n* = 178) | Controls (*n* = 712) | Matched OR (95% CI)[b] | *P* |
|---|---|---|---|---|
| Never screened | 86 (48.3) | 277 (38.9) | 1 | 0.003 |
| One | 45 (25.3) | 149 (20.9) | 1.04 (0.67–1.58) | |
| Two | 21 (11.8) | 126 (17.7) | 0.50 (0.29–0.88) | |
| Three | 19 (10.7) | 103 (14.5) | 0.52 (0.28–0.94) | |
| Four | 7 (3.9) | 57 (8.0) | 0.30 (0.12–0.76) | |

[a]Number (and percentage) of subjects who participated during each period. [b]Odds ratio and 95% CI to have a screening history compared with no screening history before the reference date.

© Cancer Research Campaign 1999

screening test in the short period preceding the diagnosis, thus explaining this apparent risk increase. In the controls, who are mostly without cancer, the screening tests are more scattered over time. When considering the 4–12 month, 13–24 month and 25–36 month segments, a statistically significant protective effect was seen. Similar findings have been observed in other studies. Wahrendorf et al (1993) considered mainly the time period 6–36 months prior to diagnosis and the effect of screening was considerably lower when the first 6-month period was included. Lazovich et al (1996) found no protective effect when considering the whole year before the reference date. The authors have shown that this result cannot be explained by the use of FOBT as a diagnostic test. Risk estimates were unchanged when the analysis was restricted to those FOBT declared to be performed in asymptomatic subjects.

As in other studies, our data indicate that the risk was lower for a 3-year period and that no reduction in risk existed when considering longer periods. Such findings lie behind current screening recommendations suggesting that FOBT should be repeated at least biennially. The risk estimate of dying of colorectal cancer associated with the number of participations in screening campaigns was decreased significantly only in those who participated in two or more screening campaigns. No mortality reduction was shown in those who participated only once, thus suggesting that a possible selective effect of participants cannot be held as an explanation for the observed mortality reduction.

We conclude that this population-based case–control study contributes further evidence that biennial faecal occult blood screening with the Hemoccult II test can reduce colorectal cancer mortality. However, because the case–control method takes no account of participation rates, allowance must be made for this factor in interpreting the results.

### ACKNOWLEDGEMENTS

This project was funded by the Europe Against Cancer Programme, INSERM, the Fonds National de Prévention, the Burgundy Regional Council and the French League Against Cancer. We would like to thank Professor Jacques Esteve for his methodological assistance and Pascale O'Sullivan for proof-reading this article.

### REFERENCES

Berrino F, Sant M, Verdecchia A, Capocaccia R, Hakulinen T and Estève J (1995) *Survival of Cancer Patients in Europe? The EUROCARE Study*. IARC scientific publications no. 132. IARC: Lyon

Hardcastle JD, Chamberlain JO, Robinson MHE, Moss SM, Amar SS, Balfour TW, James PD and Mangham CM (1996) Randomised controlled trial of faecal occult blood screening for colorectal cancer. *Lancet* **348**: 1472–1477

Kronborg O, Fenger C, Olsen J, Jorgensen OD and Sondergaard O (1996) Randomised study of screening for colorectal cancer with fecal occult blood test at Funen in Denmark. *Lancet* **348**: 1467–1471

Lazovich D, Weiss NS, Stevens NG, White E, McKnight B, Wagner EH (1995) A case–control study to evaluate efficacy of screening for faecal occult blood. *J Med Screening* **2**: 84–89

Mandel JS, Bond JH, Church TR, Snover DC, Bradley M, Schuman LM and Ederer F (1993) Reducing mortality from colorectal cancer by screening for fecal occult blood. *N Engl J Med* **328**: 1365–1371

Newcomb PA, Norfleet RG, Storer BE, Surawicz T and Marcus PM (1992) Screening sigmoidoscopy and colorectal cancer mortality. *J Natl Cancer Inst* **84**: 1572–1575

Parkin DM, Whelan SL, Ferlay J, Raymond L and Young J (1997) *Cancer Incidence in Five Continents*, Vol. VII. IARC Scientific Publication no. 143. IARC: Lyon

Saito H, Soma Y, Koeda J, Wada T, Kawaguchi H and Sobue T (1995) Reduction in risk of mortality from colorectal cancer by fecal occult blood screening with immunochemical hemagglutination test. A case–control study. *Int J Cancer* **61**: 465–469

Selby JV, Friedman GD, Quesenberry CP and Weiss NS (1993) Effect of fecal occult blood testing on mortality from colorectal cancer. *Ann Intern Med* **118**: 1–6

Tazi MA, Faivre J, Dassonville F, Lamour J, Milan C and Durand G (1997) Participation in faecal occult blood screening for colorectal cancer in a well defined French population: results of five screening rounds from 1988 to 1996. *J Med Screen* **4**: 147–151

Thomas WM, Pye G, Hardcastle JD and Walker AR (1992) Screening for colorectal carcinoma: an analysis of the sensitivity of Haemoccult. *Br J Surg* **79**: 833–835

Warhendorf J, Robra BP, Wiebelt H, Oberhausen R, Weiland M and Dhom G (1993) Effectiveness of colorectal cancer screening: results from a population-based case–control study in Saarland, Germany. *Eur J Cancer Prev* **2**: 221–227

Zappa M, Castiglione G, Grazzini G, Falini P, Giorgi D, Paci E and Ciatto S (1997) Effect of faecal occult blood testing on colorectal mortality: result of a population-based case–control study in district of Florence, Italy. *Int J Cancer* **73**: 208–210

© Cancer Research Campaign 1999
